# Influence of Nutrient Intake on 24 Hour Urinary Hydration Biomarkers Using a Clustering-Based Approach

**DOI:** 10.3390/nu12102933

**Published:** 2020-09-25

**Authors:** William M. Adams, Michael Wininger, Mitchell E. Zaplatosch, Derek J. Hevel, Jaclyn P. Maher, Jared T. McGuirt

**Affiliations:** 1 Department of Kinesiology, University of North Carolina at Greensboro, Greensboro, NC 27412, USA; mezaplat@uncg.edu (M.E.Z.); djhevel@uncg.edu (D.J.H.); jpmaher@uncg.edu (J.P.M.); 2 Cooperative Studies Program, Department of Veterans Affairs, West Haven, CT 06516, USA; Michael.wininger@yale.edu; 3 Department of Biostatistics, Yale School of Public Health, New Haven, CT 06510, USA; 4 Department of Nutrition, University of North Carolina at Greensboro, Greensboro, NC 27412, USA; jtmcguir@uncg.edu

**Keywords:** urine osmolality, fluid homeostasis, water, macronutrients, urine volume, micronutrients

## Abstract

Previous work focusing on understanding nutrient intake and its association with total body water homeostasis neglects to consider the collinearity of types of nutrients consumed and subsequent associations with hydration biomarkers. Therefore, the purpose of this study was to analyze consumption patterns of 23 a priori selected nutrients involved in osmotic homeostasis, as well as their association with 24 h urinary hydration markers among fifty African–American first-year college students through a repeated measures observation in a daily living setting. Through application of hierarchical clustering, we were able to identity four clusters of nutrients based on 24 h dietary recalls: (1) alcohol + pinitol, (2) water + calcium + magnesium + erythritol + inositol + sorbitol + xylitol, (3) total calories + total fat + total protein + potassium + sodium + zinc + phosphorous + arginine, and (4) total carbohydrates + total fiber + soluble fiber + insoluble fiber + mannitol + betaine. Furthermore, we found that consumption of nutrients in Cluster #2 was significantly predictive of urine osmolality (*p* = 0.004); no other clusters showed statistically significant associations with 24 h urinary hydration biomarkers. We conclude that there may be some nutrients that are commonly consumed concomitantly (at the day level), across a variety of settings and populations, and that a limited subset of the clustering of these nutrients may associate with body water status.

## 1. Introduction

Nutrient balance plays a critical role in human development [1,2,3] and interplays with every major organ system across the stages of life [4,5,6,7]. The nutrient matrix, in its broad conception [5,8], illustrates the importance of synergistic interactions between nutrients whose co-concentration has vital implications for prevention of injury and disease [9,10,11]. Tandem consumption of certain nutrients (both macronutrients and micronutrients) creates synergies that are critical to body functions. Therefore, it is important to study the impact of tandem consumption of nutrients on health and wellness. 

Water, often termed the “forgotten nutrient” due to its non-contributory role in caloric intake, is vital for sustaining life and is intimately involved in metabolism, substrate transport across cellular membranes to maintain cellular homeostasis, thermoregulation and cardiovascular function. In exercising settings, extensive evidence has shown the effect of total body water deficits on the degradation of physical [12] and cognitive performance [13], as well as the influence that hydration has on mitigating heat-related illness [14,15,16]. Regarding long-term health outcomes, inadequate water intake is associated with cardiometabolic and renal dysfunction, as well as chronic diseases such as diabetes, obesity, chronic kidney disease, urinary tract infections, and cardiovascular disease [17,18,19]. Given the complexities surrounding regulation of total body water regulation and homeostasis, further exploration is needed to examine the physiological processes that may influence body water regulation and homeostasis.

Nutrients may mediate and/or moderate total body water and fluid homeostasis by influencing the osmotic pathways responsible for intra- and extra-cellular fluid shifts within the body. Determining the types of nutrients that mediate and/or moderate total body water and fluid homeostasis is of continued interest within the scientific literature [20,21,22,23]. Within sport and exercise settings specifically, investigating how beverage composition optimizes total body water and restores body water deficits has been thoroughly investigated [24] and evidence shows that the addition of sodium [23,25,26], carbohydrates [27], and protein [28,29] improves water retention and restores body water lost in sweat. Outside of sport and exercise, determining which nutrients assist in optimizing water balance during everyday life may yield insights into developing interventions to target health-protective behaviors to limit long-term morbidity and mortality from preventable diseases. Yet, the interactions between nutrients and water balance at the day level remain inconclusive.

As a matter of research best-practices, there is a need to consider identifying nutrients that are consumed in consistent patterns. By finding nutrients that are consumed together, novel insights about nutritional habits, particularly in cause-and-effect relationships [30,31,32] on health-related outcomes may be garnered. Further, taking into account nutrients that are consumed together may avoid potentially corrupting a statistical analysis by inappropriately including predictor variables that are related to one another [33,34,35,36,37]. Thus, finding nutrients that are consumed together is important, whether as a building block for drawing causal inference, or as a data-conditioning step in assuring quality in a multi-variate model.

Recently, a hierarchical clustering model applied to 19 nutrients yielded 9 modules as follows: (1) glycemic load + carbohydrates + sodium, (2) protein + fat + zinc, (3) magnesium + calcium, (4) pinitol, (5) caffeine, (6) fiber + betaine, (7) water, (8) potassium + xylitol + erythritol + inositol, and (9) mannitol + sorbitol [38]. When determining the aforementioned clusters’ associations with both urinary and hematologic hydration biomarkers following a 161 km road cycling race, Muñoz et al. [38] observed that urinary hydration biomarkers immediately following and 1 h post-race were most impacted by the nutrients consumed by participants leading up to, during, and immediately following the completion of the race. The resulting nutrient clusters and the associations with hematologic and urinary hydration markers emanating from this study is unsurprising given the impetus within the sport nutrition industry to optimize the composition of sport-related nutrition products for health and performance.

By conducting a cluster-based analysis, Muñoz et al., were able to better account for the collinearity between various nutrients that are often consumed concomitantly. By consolidating the nutrients into their representative clusters, the regression models derived by Muñoz et al. [38], were properly weighted to reflect consumption patterns. Without this clustering, nutrients consumed commensurately would draw greater share of the total model variance, leading to disproportionate “leverage” within the model. For example, by comparing the consumption pattern of water (Group 7) versus the consumption pattern of ensemble-averaged data from potassium + three polyols (Group 8), these two groups are weighted equally in the analysis of their association with the markers of hydration. Without this clustering, Group 8 would have four times the representation of Group 7 in the model, a major discrepancy in the proportion of total variance. Accounting for the collinearity amongst consumed nutrients and by equally weighting the nutrient clusters may inform an improved understanding of the aforementioned relationships, which could then be used to develop targeted nutritional interventions to address topics related to human health and performance. 

While the Muñoz et al. [38] study provides a novel approach in beginning to develop an understanding how various nutrients cluster together and the resulting clusters’ association with hematologic and urinary hydration biomarkers, there are remaining gaps in the literature. First, the aforementioned study only assessed participants competing in an ultra-endurance road cycling event in hot environmental conditions, thus, the findings are limited in generalizability when compared to a broader sample population. Second, the associated impact of prolonged exercise and environmental heat stress on urinary hydration markers impedes the ability to fully understand the relationships between nutrient intake and urinary hydration biomarkers. Here, we adapt the approach taken by Muñoz et al., in application to data collected in the Freshman Reports of Eating, Exercise, Sitting and Health (FRESH) study [39]. Our specific objective was to identify hydration-related nutrients that were consumed concomitantly in a population of free-living African American emerging adults enrolled in their first semester (Fall semester) of college in the Southeastern United States. As a secondary objective, we sought to analyze the association of consumed nutrients to 24 h urinary hydration measures. Given the work of Muñoz et al., our focus was restricted to inquiry on the nutrient clusters, and not whether there were clusters of participants with similar consumption patterns. Since we sought to assess the associations between nutrient intake and urinary hydration biomarkers, we intentionally selected nutrients that contribute to osmotic homeostasis within the body: macronutrients (water, fat, protein, carbohydrate, fiber (soluble and insoluble)) [27,40,41,42,43,44,45,46,47,48,49,50,51,52], organic osmolytes (calcium, potassium, magnesium, zinc, sodium) [41,50,53,54], and other osmolytes (arginine, betaine, phosphorus, alcohol, erythritol, inositol, mannitol, pinitol, sorbitol, and xylitol) [55,56]. 

## 2. Methods

### 2.1. Participants and Design

Fifty college freshmen (70% female) participated in an observational study that took place at a large University in the southeastern United States during the Fall 2018 academic semester. Participants identifying as African American/Black on their college admissions application, enrolling in their first semester of college and between the ages of 18–25 were invited to participate in this study. Criteria excluding participants from participation were: (1) clinically relevant diseases altering normal body water regulation, (2) previous history of surgery on the gastrointestinal tract, (3) currently taking any medications that are known to influence total body water and body water regulation and (4) actively attempting to gain and/or lose weight. Further, female participants were excluded if they were currently pregnant. Prior to participation, all participants provided written and informed consent to participate. This study was approved by the University of North Carolina at Greensboro’s Institutional Review Board (#18-0269).

### 2.2. Procedures

This study was part of a larger study that utilized ecological momentary analysis to assess the relationships between physical activity and dietary intake in African–American college freshman over the course of 7 consecutive days [39]. For the purposes of this study, we are including data collected at an introductory session on day 1 and dietary recalls and urine collected on days 4, 5, and 6. At an introductory session, participants’ height and body fat percentage were assessed by a trained research assistant. Participants were also instructed on the 24 h urinary collection procedures. Participants were provided a clean container and were instructed to provide a complete urine void for all occasions occurring over a 24 h timeframe. Male participants were instructed to void directly into the clean container (10 cm diameter opening), whereas females were instructed to void into a separate graduated specimen pan from which they then poured into the clean container for storage.

Participants were instructed to begin collecting their urine on day 4 upon waking and were scheduled to arrive to the laboratory the following day (day 5) between the hours of 0600–0900 to drop off their 24 h urine sample and provide a measure of their nude body mass. Participants were provided a clean container and were instructed to repeat the same process for the next two consecutive days (days 5 and 6). Participants were scheduled to drop off their 24 h urine sample ±1 h from their first visit to ensure consistency with each 24 h urine sample. Urine collection completeness was confirmed daily by the researchers.

On days 5, 6, and 7 completed a multi-pass, telephone-based dietary recall with a research assistant to report their food and fluid intake from the day prior. Therefore, dietary recalls reflected food and fluids consumed on days 4, 5, and 6 and consisted of 1 weekend day and 2 weekdays. Throughout the study, participants were instructed to go about their normal daily routines to allow for an ecologically valid assessment of their dietary intake behaviors. 

### 2.3. Measures

#### 2.3.1. Anthropometric Variables

Height to the nearest 0.1 cm was measured using a standardized wall-mounted stadiometer (Model 216, Seca, Chino, CA, USA). Nude body mass to the nearest 0.1 kg was measured using a digital scale (WB-800S, Tanita Corporation, Tokyo, Japan). Body density was measured using the established 3-site skinfold methodology by Jackson and Pollock [57,58]. Measures for each site (men: chest, abdomen and thigh; women: suprailiac, tricep and thigh) were taken in duplicate and averaged together. To estimate body fat percent, we used the methodology established by Siri [59].

#### 2.3.2. Hydration Variables

For 24 h hydration status, each 24 h urine sample was assessed for urine volume (U_VOL_) to the nearest 0.0001 kg using a digital scale (Ranger 2000, OHAUS Corporation, Parsippany, NY, USA), urine osmolality (U_OSMO_) measured in duplicate using the freezing point depression method (Model 3320, Advanced Instruments Inc., Norwood, MA, USA), and urine specific gravity (U_SG_) using a digital refractometer (Reichert AR200, Reichert Technologies, Buffalo, NY, USA).

#### 2.3.3. Dietary Intake

Food consumption was collected via telephone interview with a trained researcher to account for all foods and fluids consumed over the course of 24 h for each of the 3 days of data collection. Food consumption was converted to nutrient weight using the Nutritional Data System for Research (NDSR) software (Nutrition Coordinating Center, University of Minnesota, Minneapolis, MN, USA) [60]. Nutrients involved in osmotic homeostasis were extracted from each participant’s 24 h dietary recall and included the following nutrients: macronutrients (water (includes water content in all foods and fluids consumed), fat, protein, carbohydrate, fiber (soluble and insoluble) [27,40,41,42,43,44,45,46,47,48,49,50,51,52], organic osmolytes (calcium, potassium, magnesium, zinc, sodium) [41,50,53,54], and other osmolytes (arginine, betaine, phosphorus, alcohol, erythritol, inositol, mannitol, pinitol, sorbitol, and xylitol) [55,56].

### 2.4. Statistical Analysis

Data from all 150 data collections (3 visits × 50 participants) were analyzed ensemble via k-means clustering. The k-means approach is a stochastic process with some probability of different results in serial repetitions of the clustering. Given this, we chose to divide the data into subsets of 20% (n = 30 randomly selected observations) and replicate the clustering of a sufficiently large number (100) of iterations. For each iteration, all 23 a priori selected nutrients were inspected for normality via the Shapiro–Wilk test with threshold for non-normality at *p* < 0.05. Nutrients whose distributions were non-normal were log-transformed following unity addition. Nutrients were then scaled to standardized interval (mean zero, unit standard deviation) and clustered via k-means where k (pre-defined number of clusters) ranged systematically from 3 to 9 (yielding a total of 100 × 7 = 700 cluster analysis iterations). 

For each iteration, each nutrient was inspected for co-clustering with each other nutrient (23 × 22 = 506 total nutrient pairings). This matrix of cluster pairings was normalized to scale 0 (no pairings) to 1 (pairings in all iterations), converted to a distance matrix, and transformed into a two-dimensional scatter of co-varying nutrients via classical multi-dimensional scaling (MDS) [61]. The optimal number of nutrients was subsequently determined through visual inspection of this MDS scatter; this cluster cardinality was retained for all downstream analysis. Given that all participants contributed the same number of replicates (three) and were all spaced evenly (24 h), neither time information nor participant ID were factored into the clustering analysis: all data were analyzed ensemble, with equal stratification and without adjustment. 

### 2.5. Modeling

Pursuant to a view of the associations between nutrients and osmotic homeostasis, we sought to measure the observed nutrient volumes as putative predictors of body water status via a generalized linear model. Our primary objective was to identify which-nutrients cluster with which-other nutrients, i.e., which nutrients are consumed in similar patterns among this population. The secondary objective of this work is to describe the association between these nutrients and markers of body water status. The novelty of this study is that the association analysis of our secondary objective (a general linear model) is informed by the outcome of our primary objective (the clustering): instead of a conventional modeling approach where each nutrient is included in the model with equal weight (and thus presumed to have fully independent patterns of variation), we acknowledge a priori that one or more of the nutrients may have some shared patterns of consumption and thus accommodate our model appropriately, reducing the relative weights of redundant nutrients, and thus giving relatively greater impact to distinct groups (clusters) of nutrients for maximum statistical power. 

Given the repeated-measures nature of the dataset, we chose a mixed effects model with the hydration markers as response variables, macronutrient clusters as fixed effects, and participant ID as a random effect. Three response variables were considered: urine volume (mL), urine specific gravity (g/cm^3^), and urine osmolality (mOsm/kg). Mixed effects models were implemented in R (v4.0.0), via the lme4 package, using default settings, i.e., maximum likelihood estimation via the restricted maximum likelihood (REML). Statistical significance in a mixed effects model can be obscure, however there is guidance that the likelihood ratio test meaningless in comparing two models with disparate fixed effects [62]. Consequently, we follow the t-as-Z approach which compares the regression coefficient’s estimated value with the estimated standard error for the coefficient, on the assumption of an underlying normal distribution.

## 3. Results

### 3.1. Descriptive Statistics

Participant demographics are shown in Table 1. Across 23 nutrients, 20 were observed sufficiently frequently as to support reporting summary statistics (Table 2); alcohol (117 zeros; 78%), erythritol (113; 87%), and pinitol (100; 67%) were reported as zero with enough frequency as to obsolesce their summarization. Across participants 24 h urinary hydration measures of U_VOL_, U_OSMO_, and U_SG_ were (mean ± SD) were 0.84 ± 0.45 L, 700 ± 236 mOsm/kg, and 1.020 ± 0.006 AU, respectively. 

Furthermore, we note that the within-patient clusters, while highly correlated (median correlation: 0.96, Interquartile Range (IQR): 0.93–0.98), the correlation of nutrient consumption across all participants was also high: 0.95 (0.90–0.97), suggesting that there is rather little specificity to individuals that isn’t already described by the group at-large. These correlations are decimated in a z-normalized dataset (indeed: in this standardized data view, the all-comers dataset shows a higher signed average correlation coefficient than the within-participant correlation.

### 3.2. Nutrient Clustering

**Figure 1:**
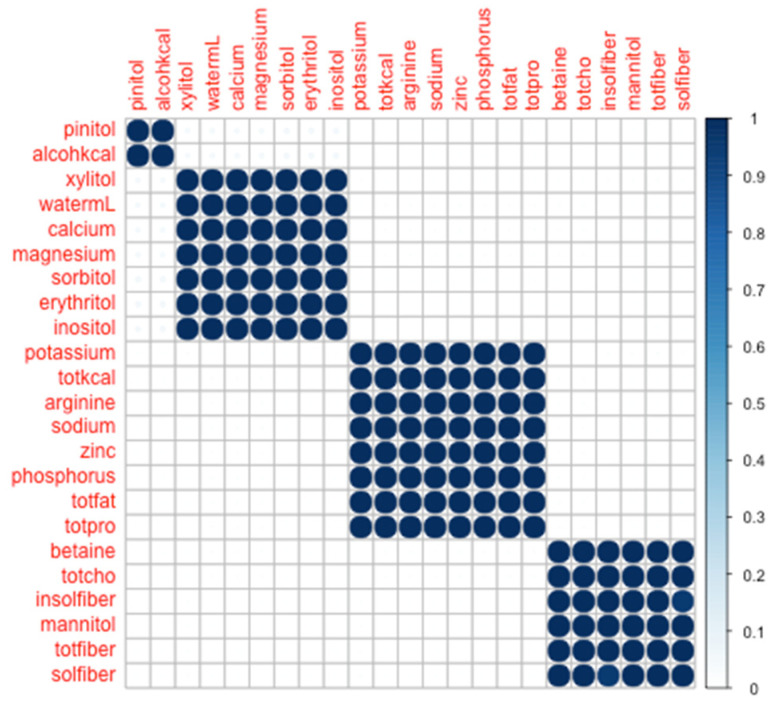
Co-clustering heat-map across 23 a priori selected nutrients consumed across a day, with four modules identified through visual inspection. Color bar shows proportion of co-clusterings across 700 iterations.

By inspection of the multi-dimensional scaling scatter, we determined that the data appeared to cluster naturally into four modules: (1) pinitol + alcohol, (2) xylitol + water + calcium + magnesium + sorbitol + erythritol + inositol, (3) potassium + total Calories + arginine + sodium + zinc + phosphorous + total fat + total protein, and (4) betaine + total carbohydrates + insoluble fiber + mannitol + total fiber + soluble fiber (Figure 1).

From Figure 1, it is evident that the co-clustering of these nutrients based on day-level consumption into four modules is robust. To wit, where an idealized co-clustering matrix would have entirely binarized cell values (0 or 1, corresponding to never co-clustering or always co-clustering, respectively), we observed 476 out of 506 nutrient pairs to have values ≤1% or ≥99%, and all nutrient pairs to be ≤5% or ≥95%. Thus, any two nutrients were either always (or nearly always) found together, or never (or nearly never) found together. 

### 3.3. Modeling

Among the three hydration biomarkers tested against the clustered macronutrients, only a single term showed statistical significance: Cluster #2 (water + calcium + magnesium + erythritol + inositol + sorbitol + xylitol) was found to be significantly and inversely associated with urine osmolality (*p* = 0.004). Further, this can be interpreted to state that the consumption of water, calcium, magnesium, erythritol, inositol, sorbitol, and xylitol was associated with a decrease in 24 h urine osmolality (i.e., improved hydration state). No other clusters were observed to be statistically significant at the even the un-corrected threshold (*p* < 0.05) for urine osmolality; no significant associations were observed for urine specific gravity, nor urine volume. 

### 3.4. Sensitivity

As an exploratory view into the effect of nutrient cluster on total body water status, we performed a cursory sensitivity analysis of our results, as above, following the addition of two additional predictors with putative explanatory value: sex and body mass index (BMI). Incorporating these terms as fixed effects into our mixed model, we find that the results for the statistical significance of the clusters did not change: the singleton significant cluster in urine osmolality remained, and none of the other clusters became significant when accounting for sex or BMI.

We do observe that sex was a significant predictor of total body water status in two models (urine volume and specific gravity as response variables): 0.01 < *p* < 0.05.

## 4. Discussion

### 4.1. Study Novelty

To our knowledge, only one other study [38] has applied a clustering approach to nutrient consumption to examine relationships with hydration biomarkers. However, this other study was performed on trained athletes as they participated in a prolonged (161 km) cycling event, and its results were narrow in scope, and left open questions about study generalizability. Here, we draw inferences about nutrient clustering and its association to 24 h urinary hydration variables in a free-living setting, with repeated measures over three days, which is much more representative of the bulk of broad inquiry in nutrition. Further, our study assessed first semester college students, which represents a unique time in one’s life; the transition to college presents with numerous lifestyle changes in that the individual often utilizes this newfound independence to develop health behaviors that may be responsible for long-term health outcomes.

Generally, the quantities of nutrients consumed in the participants observed in our study match the expectation for community dwelling young-adults. Our findings also show that the a priori selected nutrients clustered within four distinct groupings and, when modeled against 24 h urinary hydration measures, Cluster #2 (Figure 1) was the only cluster significantly associated with any of the hydration measures (urine osmolality). This is to be interpreted as the variation of intake of these nutrients (xylitol + water + calcium + magnesium + sorbitol + erythritol + inositol) reaches statistical significance in urine osmolality. Given the sensitivity of urine osmolality in quantifying the concentration of solute in a given urine sample, it is unsurprising that statistical significance was only observed when associating nutrient intake and 24 h urine osmolality. Further, based on the nutrients comprising cluster #2 (water, calcium, magnesium, erythritol, inositol, sorbitol, and xylitol), and the direction of the association between cluster #2 and U_OSMO_, the collinearity of water and these osmolytes was associated with a decrease in U_OSMO_. We also note that our participants were chronically underhydrated [63,64,65] throughout the study as shown by their total water intake (median (IQR), 1573 (1097–2072) mL) and associated 24 h urinary hydration measures. It is worthy to consider whether this result implies that Cluster #2 would suit as the singular nutrient variable to survive in a subsequent or separate modeling enterprise. This is an intriguing proposition and may well be the case, depending on context. Prima facie, we recognize that the statistical significance of this cluster, distinguishes it (and its constituent nutrients) as a target for deeper study. However, those seeking a so-called “minimal model” would be well-advised to use an objective algorithm for ruling-in or ruling-out model predictors, e.g., step-wise regression.

### 4.2. Comparability to Other Studies

Because we are unaware of other any other studies reporting nutrient clustering and the association to hydration biomarkers, we are able to compare our findings only with those reported by Muñoz et al. [38]. Comparing clustering results is made more complex by differences between the study designs. Circumstantially, our studies are quite different: Muñoz et al. [38] studied trained athletes in a strenuous competition (161 km cycling event) under challenging environmental conditions (a hot day in late summer); the FRESH study measured young adults with no expectation or assumption of fitness or physical exertion, in a repeated measures assessment in a daily-living setting. Analytically, there are substantial differences between our studies, perhaps the most important of which is the collection of different sets of nutrients: because clustering reflects the relationships between variables present in the analysis, adding or subtracting one variable influences the apparent relative strength of the associations between the other variables, altering the result. True comparability between studies would require homogenous settings, similar recruitment pools, and identical variate sets.

Nevertheless, there is value in comparing results, despite these differences. Between these two studies, the 16 common nutrients are: Protein, Fat, Fiber, Water, Betaine, Calcium, Magnesium, Potassium, Sodium, Zinc, Erythritol, Inositol, Mannitol, Pinitol, Sorbitol, and Xylitol. We observe the following similarities: Protein, Fat and Zinc; Calcium and Magnesium; Erythritol, Inositol and Xylitol; and Fiber and Betaine all cluster together in both studies. Additionally observed in both studies: Pinitol’s consumption was found to not co-cluster with any of the common nutrients, which may be associated with the types of foods consumed by both sample populations. Given the differences between the studies, these replicated results provide compelling evidence for the robustness of these co-cluster relationships, and strongly suggest that these consumption patterns might be robust to demography and setting. 

Notable discrepancies between our studies include: the relationship between sodium and potassium (Muñoz: clustered apart; here: clustered together), mannitol and sorbitol (Muñoz: a cluster unto themselves; here: clustered apart), and water (Muñoz: Water clustered by itself; here: water co-clustered with many other nutrients). Whether these differential results are due to veridical differences in consumption patterns between the two studies, or due to the (modest) differences in analytical approach, or a “passive” consequence of the two datasets containing mutually distinct nutrients (Muñoz: glycemic load, carbohydrates and caffeine; presently: total caloric intake, total carbohydrates, alcohol, phosphorous, soluble fiber, insoluble fiber, and arginine) is unknown. We note also that Muñoz et al. measured participants cross-sectionally, whereas the present study is of a repeated measures design. We considered transforming our empirical data into a representational dataset comprising a single datum per participant (average of all three days per-participant), but determined that this would be potentially lossy, obscuring day-to-day fluctuations in nutrient intake and body-water status. By muting the nuances of the dataset in this way, we would not only decrease sample size, but might lose valuable signal. While three-day means are canon, we felt that our analysis was enhanced by embracing the inherent variability in the full dataset. 

Muñoz et al. [38] found that urinary hydration biomarkers collected immediately post and 1 h post a 161 km road cycling race (mean finish time, 374.9 ± 74.8 min) were significantly associated with nutrient intake across multiple clusters formed in their respective clustering analysis. This is in contrast to our findings, where we identified only one cluster to be associated with any one of our 24 h urinary hydration measures (U_OSMO_). It is speculated that these differences are due to the respective methodological designs of each study. The former study was conducted surrounding a 161 km road cycling race and included a designed water bolus intervention between the immediate post-race measures and 1 h post-race measures. The current study was observational in design and conducted on a free-living population of first semester college students. Additionally, we collected a 24 h urine sample from all participants, which provides a more accurate assessment of daily hydration status compared to the spot samples [66] that were collected in Muñoz et al. [38]. Further, the impact that exercise and environmental heat stress has on urinary hydration indices [66,67] must not be discounted when discerning potential differences between these two studies.

### 4.3. Interpretation

Here, we clustered nutrients consumed by 50 individuals. We can interpret a given cluster as identifying one or more nutrients that appear to co-vary in similar ways (e.g., when one nutrient appears in a relatively high quantity, the other nutrient appears in a relatively high quantity). This relationship can also be inverse: as one nutrient appears to increase, the other nutrient appears to decrease (and vice-versa). Any such coupling would be detected and would cluster together. By gaining a better understanding of these relationships, we can begin to garner a more thorough assessment of dietary intake in college students, which can then be used to drive institutional and student-specific interventions to promote healthy dietary behaviors. 

It is important to emphasize that the inferences that can be drawn from this analysis are at the level of nutrient, not of participant. The consumption patterns were viewed across 150 records (50 participants × three replicates). By constructing the dataset in this way, we had a rich dataset from which to construct the consumption of a comparatively small number of nutrients, expressed through a comparatively large number of observations. 

It is well acknowledged that increasing total water intake over a 24 h period decreases 24 h urine osmolality and vice versa when total water intake is decreased [64,68]. It is not surprising to observe a statistically significant relationship between 24 h urine osmolality and cluster #2 (xylitol + water + calcium + magnesium + sorbitol + erythritol + inositol) in our study given the free-living conditions in which our participants were observed and the impact that water and the other osmolytes clustered in cluster #2 has on total body water balance and homeostasis. It is interesting to note that there were no other statistically significant relationships between cluster #2 and the other 24 h urinary hydration measures. However, we speculate that this is due to the sensitivity of these urinary hydration measures; urine osmolality is a more sensitive measure of hydration status compared to urine volume, urine specific gravity, and urine color [65,69].

While the collinearity of some of the nutrients was expected in our clustering analysis, the lack of collinearity of other nutrients was noted. For example, we expected total carbohydrates and sodium to cluster together as glucose stimulates sodium transport within the small intestines [70], however, this was not observed within our analysis. While we cannot fully explain this lack of collinearity within this dataset, this may be due to the types, availability, and individual selection of foodstuffs of our participants (e.g., university cafeteria, participant-prepared foods, restaurants in close proximity to the university, etc.). Conversely, and as expected, we observed magnesium and calcium to form a cluster with water and other short-chain carbohydrates. This can be attributed to the role that magnesium and calcium and the organic osmolytes xylitol, sorbitol, erythritol and inositol have in promoting water retention and total body water balance [71]. Further, since our participants consumed water well below the recommended daily adequate intake volumes set forth by the European Food Safety Authority (2.0 and 2.5 L/d for females and males, respectively) [64] and the Institutes of Medicine (2.9 and 3.7 L/d for females and males, respectively) [72], the co-consumption of these nutrients may have been associated with maintaining body water homeostasis. It is unclear as to how increasing water intake, and thus, altering 24 h urinary hydration measures, would affect the aforementioned relationships and warrant further inquiry.

**Figure 2:**
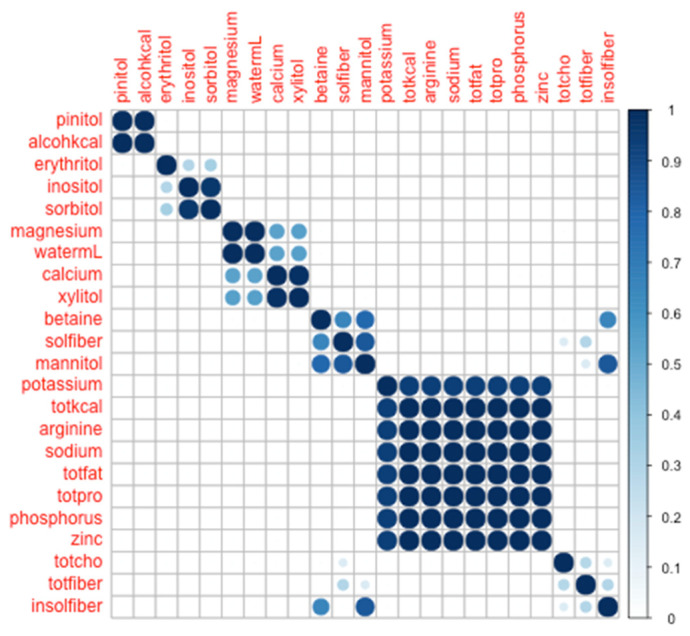
Co-clustering heat-map across 23 nutrients, forced into 9 modules.

Given that a clustering strategy is inherently data-driven, any analysis of nutrient consumption patterns is highly labile to the specific nutrients documented. Conceivably, documenting one-more, one-less, or one-different nutrient could alter the apparent relationships between the nutrients, and yield a different clustering result. Furthermore, clustering is an inherently reductionistic technique, converting a continuous measure (distance in a variable space) into categorical outcomes (nutrients in a set of clusters). Because of this discretization, some information is lost. For example, sodium and potassium were found to co-cluster in our study, but not in Muñoz et al.’s study [38]. Because Muñoz et al. reported nine distinct clusters (versus four, presently), it is possible that potassium and sodium would be seen in Muñoz’s study to cluster together, had the number of clusters been fixed at four. In this spirit, we tested whether potassium and sodium would remain co-clustered when forced to nine modules. As can be seen in Figure 2, potassium and sodium remained co-clustered, in persistent contrast to Muñoz et al.

Whether this finding suggests a fundamental difference in the consumption patterns, or (as above): artefact due to differences in data collection or analytical approach, cannot be tested conclusively in this study, and remains an attractive target for future work. Lastly, we acknowledge the complexities of interpreting analysis of replicate data as collected in this study. Some aspects of our protocol enhance the independence of the observations, e.g., collecting on one weekend and two weekdays, and the diversity of food options available on campus; some aspects of our protocol may constrain independence, e.g., the stereotyped taste preferences or consumption behaviors of each participant. While it is impossible to assess the true independence (or not-independence) of these replicates, we make no claim regarding their independence. Nevertheless, because each participant contributed the same number of replicates, in the same time interval, including the same outcome measures, and our analysis was configured to draw inferences about the nutrients, not the participants, we believe the impact of a not-fully-independent dataset is substantially mitigated.

### 4.4. Limitations

The FRESH study operated at a single college campus. The institution is large (more 20,000 undergraduates with a large menu of available food options) and situated in a metropolitan area with lots of retail dining and grocery options available, suggesting that there was a large variety of foodstuffs available. However, without exact and comprehensive data documenting food source, it is impossible to conclusively determine that the nutrient patterns observed here reflect a free and unrestricted diet, or whether there is a systematic bias introduced by observing first-year students whose dietary options may be commonly shared. Our assessment of dietary intake was also conducted at the day level where nutrients consumed over a 24 h period were measured; thus, it is difficult to determine the clustering of nutrients at the momentary level (i.e., each meal or eating occasion). Additionally, the nutrient patterns observed in this study may not accurately depict actual dietary intake given the limitations regarding 24 h dietary recalls and the known under and/or over-reporting of energy intake [73].

Furthermore, given the demographic of participants (a convenience sample of healthy emerging adults attending university), African American men and women, that were purposefully selected for this study and the known differences in 24 h urinary hydration measures when compared to non-Hispanic White college students [74], our study is limited in its generalizability. However, studying a population that is largely underrepresented in research and at higher risk for obesity and obesity-related chronic conditions at this critical stage in life provides preliminary insights into dietary patterns and warrants further investigation to determine impacts of these patterns on long-term health outcomes. It is worthy to note that publicly available reports of the institution’s diversity profile indicate 28% of the student body (approximately 5600 students) identifying as African American [75]. Given that study participation was voluntary and that no data was collected from members of the community at-large, we are strictly unable to test whether our sample was representative of the broader population. Nevertheless, we disclaim that our results, as reported here, are specific to the inferential population, nor do we suppose they generalize beyond the participant pool. Rather, we believe our findings to be interesting in their own right, and useful as a first insight into the nature of the nutrients themselves, without assumption as to the consumption patterns within or beyond any demographic group.

There are some other noted limitations that may have influenced the results of this study. Since this study was in free-living persons, we did not control for female’s menstrual cycle phase, nor control for any contraceptive use. Given the existing literature showing the impact of female sex hormones on body water regulatory hormones (e.g., arginine vasopressin), the 24 h urinary hydration measures may present with added variability among female participants who may have been in either the follicular or luteal phase of their menstrual cycle. We screened participants for current prescribed medications that may alter body water regulation, however, if participants chose not to self-repot medication use, it could have influenced our hydration assessment measures. On each day of urine collection, a researcher confirmed with the participant that they had collected all of their urine over the previous 24 h period. However, since this was a free-living observational study, and participants were free to go about their normal daily life, there may have been a discrepancy between the participant’s confirmation of 24 h urine collection and actual urine collection. Based on the retrospective analysis of the data and comparing participant’s total water intake to their 24 h urine volume, we are confident that there are no gross discrepancies in actual urine collection versus self-reported collection confirmation. Lastly, we cannot discount potential differences in total energy intake, and thus nutrient intake, in larger (i.e., greater body mass) individuals compared to smaller (i.e., lower body mass individuals), as well as the bioavailability of nutrients consumed and the impact on our results.

### 4.5. Future Work

Given the importance of replicable methodologies in scientific inquiry, it is incumbent that future study designs accommodate collection of a sufficiently large number of common-interest nutrients to allow for testing identical nutrient sets in a variety of different settings. Analytically, there is value in both heuristically determining the optimal number of clusters (here: through visual inspection) and fixing to a set number of clusters (here: through manually setting the cluster cardinality to nine) in order to support direct comparison to another study. Moreover, while we applied our methodologies to inquiry focused on nutrient clusterings, there is opportunity to deliver insight into other dimensions of nutritional study. For instance, are there clusterings among individuals: were a clustering analysis to be applied to our own cohort, would we discover phenotypes among our convenience-sampled participants; were a clustering analysis to be applied to a participant pool comprising community-dwellers, athletes, or burdened patients, would there be ability to discriminate among these cohorts? These concepts are far beyond the scope of the present paper, but provide enticing targets for next-steps research enterprise.

We might also suggest that there is value in a sensitivity analysis: how do results change with the addition or subtraction of nutrients; how do results change if water is excluded from the clustering? With more subtlety, we consider whether retaining water, but stratifying, e.g., high versus low water consumption, might impact our outcomes. Dichotomizing water in this way would decrease the power of the clustering analysis, which is presently assessing water as a continuous measure. Stratifying at the point of the regression would be a more conventional approach, but again, there is some power lost in converting a continuous variable to a dichotomous variable, and there is the additional complication that the regression would not contain water as an isolated variable, but rather as one of several nutrients in a cluster. We recognize fully the interest in assessing water by itself, and as a parameter with many interesting configurations. We believe that these approaches would make for fascinating additional papers. Naturally, there is opportunity for sharing of raw data between research groups so that distinct datasets of common nutrients can be compared directly, using identical analytical approaches. It is also prudent to utilize these analytical approaches in future work assessing the role of dietary intake to physiologic outcomes (e.g., body water regulation and homeostasis), health outcomes and risk of disease as this may afford a more thorough understanding of these relationships and drive the development of targeted treatment interventions.

## 5. Conclusions

Within the study population, first semester African American college freshman, there may be some nutrients that are commonly consumed concomitantly (at the day level), and that a limited subset of the clustering of these nutrients may associate with 24 h urinary hydration biomarkers.

## Figures and Tables

**Table 1:** Participant Demographics.

Measure	Mean (SD)
Anthropometric Measures	
Age (y)	18 (0)
Height (cm)	167 (8)
Mass (kg)	72.3 (19.8)
Body Fat (%)	22.7 (10.8)
Physical Activity	
Sedentary Time (min)	725.6 (173.1)
Light Physical Activity (min)	573.9 (124.4)
MVPA (min)	4.9 (10.2)
Sleep	
Total Sleep Time (min)	433.1 (123.9)
24 h Urinary Hydration Measures	
Urine Volume (L)	0.9252 (0.4305)
Urine Osmolality (mOsm/kg)	672 (211)
Urine Specific Gravity (AU)	1.020 (0.013)

MVPA = moderate to vigorous physical activity.

**Table 2:** Summary statistics of measured macronutrients and micronutrients among 150 study visits; count of zero-values in parentheses.

Macronutrient	Median (IQR)	Macronutrient	Median (IQR)
Alcohol (kCal, 117)	NA	Sodium (mg, 0)	3375 (2316–4260)
Arginine (g, 0)	3.1 (2.2–4.1)	Solfiber (g, 0)	3.7 (2.4–5.3)
Betaine (mg, 0)	99 (63–153)	Sorbitol (g, 20) *	16.5 (3.0–105.2)
Calcium (mg, 0)	678 (436–970)	Xylitol (g, 30) *	2 (1–7)
Erythritol (g, 133)	NA	Zinc (mg, 0)	7.0 (5.2–9.6)
Inositol (g, 0) *	123 (73.2–218.8)	Water (mL, 0)	1573 (1097–2072)
Insolfiber (g, 0)	6.0 (4.1–10.3)	—	
Magnesium (mg, 0)	182 (142–251)	Total Carb (g, 0)	199 (153–291)
Mannitol (g, 7) *	80.5 (23.2–182.5)	Total Fat (g, 0)	66.9 (49.1–95.4)
Phosphorous (mg, 0)	934 (693–1177)	Total Fiber (g, 0)	10.2 (6.8–15.7)
Pinitol (g, 100)	NA	Total Calories (kcal, 0)	1707 (1315–2198)
Potassium (mg, 0)	1572 (1091–2229)	Total Protein (g, 0)	61.7 (45.7–81.6)

Zero-intensive macronutrients, with >30% zero values, reported as NA. * = Quantities multiplied by 1000 in reporting. IQR = Interquartile Range.
